# One-step preparation of RGO/Fe_3_O_4_–FeVO_4_ nanocomposites as highly effective photocatalysts under natural sunlight illumination

**DOI:** 10.1038/s41598-022-10542-z

**Published:** 2022-04-21

**Authors:** Qana A. Alsulami, A. Rajeh, Mohammed A. Mannaa, Soha M. Albukhari, Doaa F. Baamer

**Affiliations:** 1 Chemistry Department, Faculty of Science, King Abdulaziz University, Jeddah, Saudi Arabia; 2 Physics Department, Faculty of Science, Amran University, Amran, Yemen; 3 Chemistry Department, Faculty of Science, Amran University, Amran, Yemen

**Keywords:** Environmental sciences, Chemistry, Materials science

## Abstract

The study used a one-step hydrothermal method to prepare Fe_3_O_4_–FeVO_4_ and xRGO/Fe_3_O_4_–FeVO_4_ nanocomposites. XRD, TEM, EDS, XPS, DRS, and PL techniques were used to examine the structurally and morphologically properties of the prepared samples. The XRD results appeared that the Fe_3_O_4_–FeVO_4_ has a triclinic crystal structure. Under hydrothermal treatment, (GO) was effectively reduced to (RGO) as illustrated by XRD and XPS results. UV–Vis analysis revealed that the addition of RGO enhanced the absorption in the visible region and narrowed the band gap energy. The photoactivities of the prepared samples were evaluated by degrading methylene blue (MB), phenol and brilliant green under sunlight illumination. As indicated by all the nanocomposites, photocatalytic activity was higher than the pure Fe_3_O_4_–FeVO_4_ photocatalyst, and the highest photodegradation efficiency of MB and phenol was shown by the 10%RGO/Fe_3_O_4_–FeVO_4_. In addition, the study examined the mineralization (TOC), photodegradation process, and photocatalytic reaction kinetics of MB and phenol.

## Introduction

The world of today is seeking state-of-the-art technologies to deal with the major challenges of the environment pollutions^1–3^. The fast industry expansion and development, water becomes contaminated, carrying significant concentrations of hazardous and dangerous contaminants such as dyes, inks, pesticides, and so on^4–6^. Almost 10–15% of dyes are released in the environment during the dying process, contaminating sewage water^7^. Many attempts were taken in order to use new and various light sources and nanomaterials for removal of organic pollutants by photodegradation method^7–10^. Different approaches like doping and compositing used to enhance the structural properties and photodegradation efficiency of the prepared nanomaterials. Various modified metal oxide such as, Vanadium doped CaTiO_3_^11^, Rh doped SrTiO_3_^12^, Rhodium doping barium titanate^13^, Graphene Wrapped SrTiO3 Nanocomposite^14^, … etc. have been prepared by different approaches and applied as efficient photocatalysts.

Meta-vanadates, which are characterized as cohesive materials' class and have prospective uses in various fields, are one of former mentioned materials^1,15^. In addition, because of its various stable oxidizing states (+ 2 to + 5), vanadium interacts with numerous components. However, FeVO_4_ gave low photocatalytic activity, low optical absorption and poor transport of photogenerated charges which limited its applications^7,16^. Many novel composites are created as a result of this combining procedure. Fe_3_O_4_–FeVO_4_ in particular stands out for its broad range of beneficial properties^17^. It has been investigated for distinctive applications due to its advantageous properties, including ease of preparation, low cost, environmental friendliness, and small band gap^17,18^. The present investigation could not be carried out without Fe_3_O_4_–FeVO_4_ feature of small band gap (2.7–2.03 eV)^1,19^. Furthermore, Fe_3_O_4_–FeVO_4_ has the necessary conductivity and energy levels to format nanocomposite catalysts^15,17^. In addition, it was revealed that Fe_3_O_4_–FeVO_4_ catalysts were effective applied in the treatment of various organic contaminants present in water over a wider pH range. A varying of methods were applied for water purification^20^. The photocatalysis method does not entail an issue of waste disposal. Furthermore, a lot of composite materials were prepared with graphene and the resultant composites displayed different advantages and applied in various sectors. For example, various composites including FeVO_4_·xH_2_O/Graphene^21^, ZnO/FeVO_4_^22^, FeVO_4_/Bi_7_O_9_I_3_^23^ rGO-FeVO_4_^15^, RGO–ZnWO_4_–Fe_3_O_4_^24^, N-doped RGO-FeWO_4_/Fe_3_O_4_^25^ BaWO_4_/NRGO–g-C_3_N_4_^26^, and RGO–ZnWO_4_^14^ have recently been prepared and applied in different sectors.

In addition, RGO-based ortho-vanadates were applied as useful material for water^20^. In this research, Fe_3_O_4_–FeVO_4_ and xRGO/Fe_3_O_4_–FeVO_4_ with various RGO quantities were prepared. The hydrothermal technique was used to efficiently decrease GO to RGO. The study was investigated the influence of RGO quantity on the structural characteristics and photocatalytic effectiveness of Fe_3_O_4_–FeVO_4_. The photocatalytic activities of the prepared samples were examined by the photodegradation of MB, phenol and brilliant green (BG) solution under natural sunlight illumination. The xRGO/Fe_3_O_4_–FeVO_4_ demonstrated excellent charges separation, stability, reusability, and photocatalytic activity, indicating that it is a potential material for a variety of environmental applications.

## Experimental

### Preparation of GO

Graphene oxide (GO) was prepared as described in our previous literature^27^.

### Preparation of Fe_3_O_4_–FeVO_4_ and xRGO/Fe_3_O_4_–FeVO_4_ nanocomposites

The hydrothermal technique was used to produce Fe_3_O_4_–FeVO_4_ and xRGO/Fe_3_O_4_–FeVO_4_ nanocomposites. According to this technique, 3 mmol of Fe (NO_3_)_3_·9H_2_O was dissolved in 10 ml of HNO_3_ solution with the concentration of 1 mol/L. 3 mmol NH_4_VO_3_ (Sigma-Aldrich) was dissolved in 10 ml of deionized water and kept at ultrasonic bath @50 °C for 30 min. After that, the solution of Fe (NO_3_)_3_ was added into NH_4_VO_3_ solution under vigorous agitation for 1 h. A certain quantity of the prepared GO was suspended in 20 ml of ethanol solution (2:1) under vigorously stirring and ultrasonicated for 1 h and then added to the mixture under magnetic agitation for 2 h. Finally, the resultant mixture was transferred to an autoclave with 50 ml volume and kept at 200 °C for 2 h. In comparison, a similar technique was used to produce pure Fe_3_O_4_–FeVO_4_ without RGO.

### Characterization methods

Crystal structure of the prepared samples was characterized by X-ray diffraction (XRD, Bruker-D8-AXS diffractometer, Germany) with Cu-Kα radiation at a setting of 40 kV and 150 mA. The images were captured using a transmission electron microscope (TEM) with a Jeol JEM-1230 apparatus operating at 120 kV. With the same EDAX detector, an energy-dispersive X-ray (EDx) study was performed (SEM, Hitachi S-4200). The chemical compositions (Axis Ultra DLD, Kratos) were performed using X-ray photoelectron spectroscopy (XPS) with a 325 nm excitation wavelength. At room temperature, UV–visible absorption studies were conducted by the UV‒Vis 2450 (Shimadzu) spectrophotometer to record diffuse reflectance spectra (DRS). The photoluminescence (PL) spectra were carried out with a fluorescent spectrophotometer (HORIBA-Jobin-Yvon).

### Photocatalytic activity study

#### Photodegradation studies

The photocatalytic activity of the produced catalysts was assessed for MB, phenol and BG photodegradation. Natural sunshine provided irradiation, and the reactor was encased in a water-cooling system. The solution was transported to the photoreactor after 0.05 g of sample powder was added to 50 ml of pollutant (Co = 10 MB/L). The degradation of MB, phenol and BG in solar light was performed on sunny days between 11.00 a.m. and 2.00 p.m. with a maximum temperature of 35 °C. The intensity solar light was measured every 30 min over LT Lutron LX-10/A digital Lux meter and the average light intensity was nearly constant during the experiments. The mixture was first agitated in the dark for 30 min to achieve the adsorption–desorption equilibrium. The mixture was then stirred on a magnetically under direct sunshine lighting. The degradation of MB, phenol and BG was calculated using the Eq. (1)^28^, and the change in pollutant concentrations was measured using a Shimadzu, MPC-2200 UV–Vis spectrophotometer.

1 $$ {\text{D}}\% = \left( {\frac{{{\text{C}}_{{\text{o}}} - {\text{C}}_{{\text{t}}} }}{{{\text{C}}_{{\text{o}}} }}} \right) \times 100\% $$

where C_o_ and C_t_ represent the concentrations dyes before and after irradiation, respectively (t). The reactive radicals that might be formed in photocatalytic processes were also investigated employing several scavengers at concentrations of 1 mM, including benzoquinone (BQ), isopropanol (IPA), and Na_2_EDTA as ^·^O_2_^**−**^, ^·^OH, and h^+^ scavengers, respectively^29–31^. The total organic carbon (TOC) was measured using a Shimadzu 5000 TOC Analyzer which applied to investigate the mineralization of MB, phenol and BG. After photodegradation, the %TOC of MB, phenol and BG was estimated using the following equation:

2 $$ \% {\text{TOC}} = \left( {\frac{{ {\text{TOC}}_{{{\text{Initial}}}} - {\text{TOC}}_{{{\text{Final}}}} }}{{{\text{TOC}}_{{{\text{Initial}}}} }}} \right) \times 100 $$

## Results and discussion

### XRD analysis

**Figure 1:**
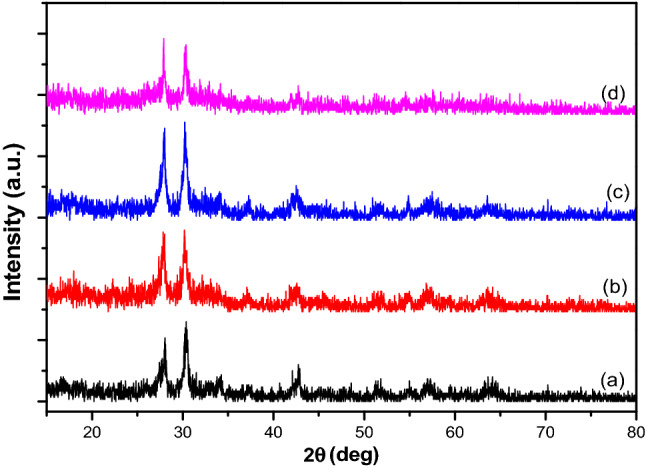
XRD patterns of (**a**) Fe_3_O_4_–FeVO_4_ and (**b**) 5%, (**c**) 10%, (**d**) 15%RGO/Fe_3_O_4_–FeVO_4_ nanocomposites.

The XRD patterns of Fe_3_O_4_–FeVO_4_ and xRGO/Fe_3_O_4_–FeVO_4_ nanocomposites are displayed in Fig. 1. The diffraction peaks of Fe_3_O_4_–FeVO_4_ demonstrates that the sample have a triclinic phase (JCPDS card No. #71–1592)^3,19,32,33^. Further, another diffraction peaks appeared at 18.51°, 30.24°, 35.61° and 73.91° matched very well with the Fe_3_O_4_ structure (JCPDS No. 19-0629)^34^.

The XRD patterns of xRGO/Fe_3_O_4_–FeVO_4_ showed similar peaks as of Fe_3_O_4_–FeVO_4_. Moreover, the sample with 15 wt.% of RGO displayed a small peak appeared at 2θ = 26.05° indicating the existence of RGO^21,35^. As seen in Fig. 1S displays positional shift of the peaks after the addition of RGO. This resulted from effect the introduction of RGO which led to changes in the lattice parameters of Fe_3_O_4_–FeVO_4_ (d-spacing changed from 3.17 to 3.19 Å at 2*θ* = 27.9°). In addition, Fig. 1 illustrates that the intensity of characteristic peaks increased with the percentage of RGO increased.

The crystal size of the prepared samples was calculated according to the Scherrer’s equation^36,37^. Table 1 illustrates that the crystals size of xRGO/Fe_3_O_4_–FeVO_4_ increased with the percentage of RGO increased compared to pure Fe_3_O_4_–FeVO_4_ indicating the existence of RGO enhanced the crystals growth of Fe_3_O_4_–FeVO_4_.

**Table 1:** Crystallite size and BG energy of the prepared photocatalysts.

Sample name	Crystallite size D (nm)	Band gap energy (eV)
Fe_3_O_4_–FeVO_4_	72.9	2.07
5%RGO/Fe_3_O_4_–FeVO_4_	79.6	2.04
10%RGO/Fe_3_O_4_–FeVO_4_	88.1	1.9
15%RGO/Fe_3_O_4_–FeVO_4_	85.8	1.98

### TEM and EDX analysis

**Figure 2:**
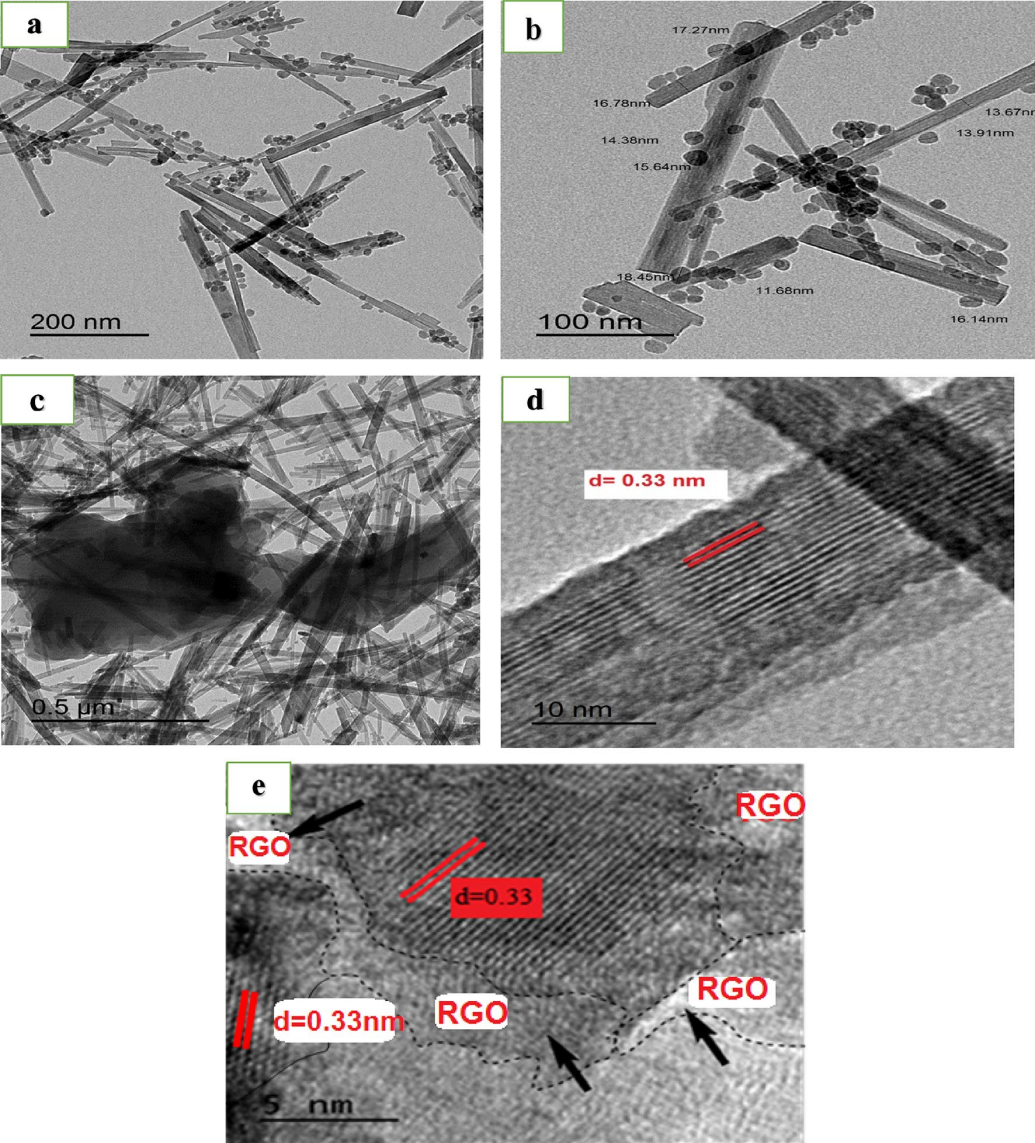
TEM and HR-TEM images of (**a**, **b**, **d**) Fe_3_O_4_–FeVO_4_ and (**c**, **e**) 10%RGO/Fe_3_O_4_–FeVO_4_.

The morphology and size of Fe_3_O_4_–FeVO_4_ and xRGO/Fe_3_O_4_–FeVO_4_ were characterized by TEM as depicted in Fig. 2. Figure 2a, b reveals that the Fe_3_O_4_–FeVO_4_ have nanorod and spherical particles structures. The particles with nanorod structure indicate to the structure of Fe_3_O_4_–FeVO_4_ while the spherical particles attribute to the Fe_3_O_4_. On the other hand, the particles with nanorods structure showed average size ~ 138 nm and ~ 20 nm in length and diameter while the spherical particles is 13.8 nm. Figure 2c illustrates the influence of the addition of reduced graphene oxide on the structure of Fe_3_O_4_–FeVO_4_. TEM images of 10%Fe_3_O_4_–FeVO_4_ (Fig. 2c) displays that the particles still have the nanorods shape. The HR-TEM images of Fe_3_O_4_–FeVO_4_ and 10%RGO/Fe_3_O_4_–FeVO_4_ are shown in Fig. 2c, e and displays that the d-spacing are 0.30, 0.32 and 0.33 nm where 0.30 nm approach to the (220) lattice plane of Fe_3_O_4_ (peak at 2ϴ = 30.24°) while 0.32 nm belong to (1–12) plane of FeVO_4_ (2ϴ = 27.9°). The increasing in the lattice spacing from 0.30 of Fe_3_O_4_–FeVO_4_ to 0.32 nm of 10%RGO/Fe_3_O_4_–FeVO_4_ resulted from effect the introduction of RGO as described in the XRD. In addition, Fig. 2e also reveals the formation of heterojunction interface between FeVO_4_ and Fe_3_O_4_ on the RGO surface.

**Figure 3:**
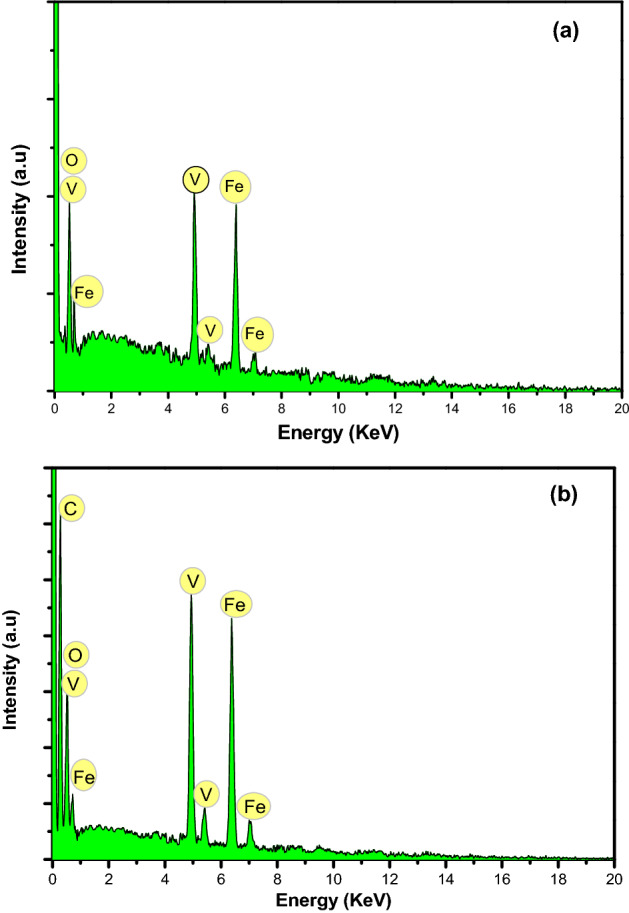
EDX spectra of (**a**) Fe_3_O_4_–FeVO_4_ and (**b**) 10%RGO/Fe_3_O_4_–FeVO_4_ nanocomposite.

Figure 3 shows the EDX spectra of Fe_3_O_4_–FeVO_4_ and xRGO/Fe_3_O_4_–FeVO_4_. Figure 3a displays that the Fe_3_O_4_–FeVO_4_ have Fe, V, C and O and no another impurities were detected. In addition, the 10%RGO/Fe_3_O_4_–FeVO_4_ nanocomposites (Fig. 3b) showed the similar elements as Fe_3_O_4_–FeVO_4_ and C which attributes to RGO in the sample. Also, Table 1S illustrates the weight percent of elements in the 10%RGO/Fe_3_O_4_–FeVO_4_ which confirmed the successful preparation of the desired nanocomposites.

### XPS studies

**Figure 4:**
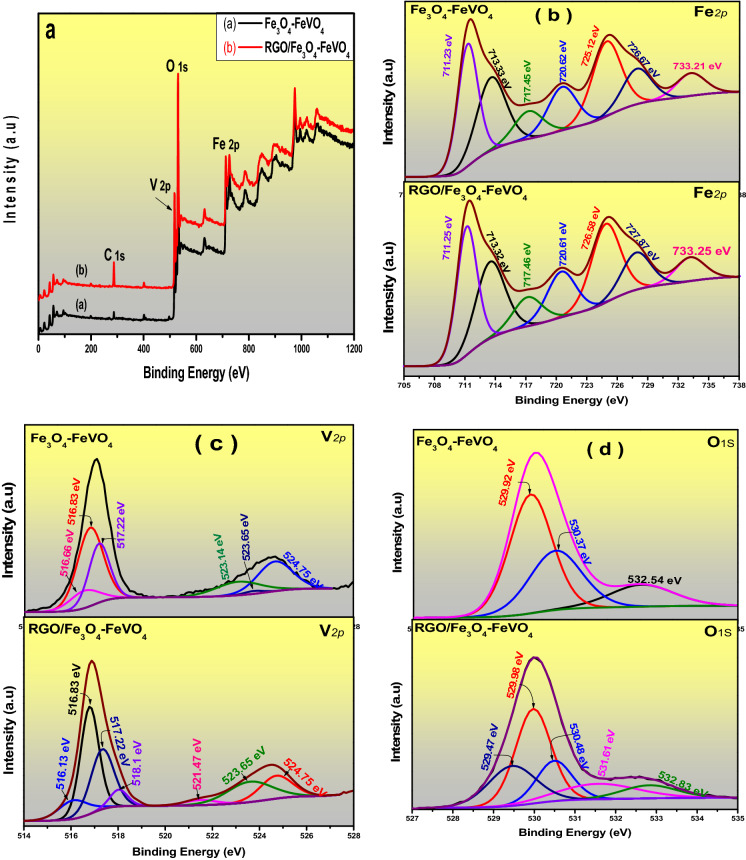
XPS spectra of Fe_3_O_4_–FeVO_4_ and 10%RGO/Fe_3_O_4_–FeVO_4_ nanocomposites.

Figure 4 displays the XPS results of Fe_3_O_4_–FeVO_4_ and 10%RGO/Fe_3_O_4_–FeVO_4_. The XPS spectrum of Fe_3_O_4_–FeVO_4_ in Fig. 4a presents peaks corresponding to Fe 2*p*, V 2*p*, and O 1*s* and the spectrum of 10%RGO/Fe_3_O_4_–FeVO_4_ showed the same peaks as Fe_3_O_4_–FeVO_4_ and C 1*s* in agreement with EDX results (Fig. 3).

The high-resolution spectra of Fe 2*p*, V 2*p*, O1*s* and C 1*s* are illustrated in Fig. 4b–d. As seen in Fig. 4b, Fe_3_O_4_–FeVO_4_ displayed main binding energies appeared at 711.23 eV and 725.12 eV belongs to Fe 2*p*_3/2_ and Fe 2*p*_1/2_ respectively. Also, the satellite peak appeared at 717.45 eV assigned to Fe^3+^ in the sample^1,21,38^. Another peaks observed at 713.33 and 726.86 eV accompanied with its satellite appeared at 720.62 and 733.21 eV which confirmed the presence of Fe^2+^^21^. Comparing with Fe_3_O_4_–FeVO_4,_ 10%RGO/Fe_3_O_4_–FeVO_4_ displayed the same main binding energies as shown in Fig. 4b. Also, the intensity peaks of 10%RGO/Fe_3_O_4_–FeVO_4_ are higher than that of Fe_3_O_4_–FeVO_4_. Figure 4c shows the high-resolution spectrum of Fe_3_O_4_–FeVO_4_. In this spectrum, six peaks were observed at 516.66, 516.83, 517.22, 523.14, 523.65 and 524.75 eV belong to V 2*p*_3/2_ and V 2*p*_1/2_ peaks. The positions of these peaks donating the existence of V^4+^ and V^5+^ of V^16,32,39^. New peaks located at 518.13 eV were observed in the spectrum of 10%RGO/Fe_3_O_4_–FeVO_4_ (Fig. 4c) comparing with Fe_3_O_4_–FeVO_4_. Also, Fig. 4c displays that the peaks located at 516.66 and 523.65 eV were shifted to 516.13 and 521.47 eV, respectively indicating the presence V^3+^ resulted due to the reduction of V^4+^ to V^3+^ state^23,40^. The formation of V^4+^ and V^3+^ in the sample indicates to existence of oxygen vacancies (Vo) in their crystal structure^23^.

Figure 4d illustrates the spectrum of O 1*s* of Fe_3_O_4_–FeVO_4_ showed three peaks located at 529.92, 530.37 and 532.54 eV assign to the lattice O in Fe_3_O_4_–FeVO_4_^23,41^. However, 10%RGO/Fe_3_O_4_–FeVO_4_ exhibited new peaks located at 529.46 and 531.66 eV attributed to the oxygen and hydroxyl groups on the RGO^2,25,39^. Also, Fig. 4d shows that the binding energies peaks of O 1*s* in the 10%RGO/Fe_3_O_4_–FeVO_4_ were shifted to 529.97, 530.47 and 532.84 eV, indicating the existence of high Vo formed after the addition of RGO^17^. Figure 2S gives compression between GO and RGO. In the XPS spectrum of GO (Fig. 2S), four peaks appear at 284.59, 286.28, 288.33 and 289.06 eV are attributed to *sp*^2^ and *sp*^3^ carbon^20,42,43^. However, positional shift of the peaks located at 288.33 and 289.06 eV to 287.84 and 288.91 eV in the 10%RGO/Fe_3_O_4_–FeVO_4_ were observed and the intensity of peaks that attributed to oxygenated groups were decreased sharply which indicates the effective reduction of GO to RGO^20,44^. Based on the results above the shifting in the peaks position of Fe 2*p*, V 2*p* and O1*s* in the 10%RGO/Fe_3_O_4_–FeVO_4_ attribute to the strong interactions between Fe_3_O_4_–FeVO_4_ and RGO in the nanocomposites^2,23^.

### DRS analyst

**Figure 5:**
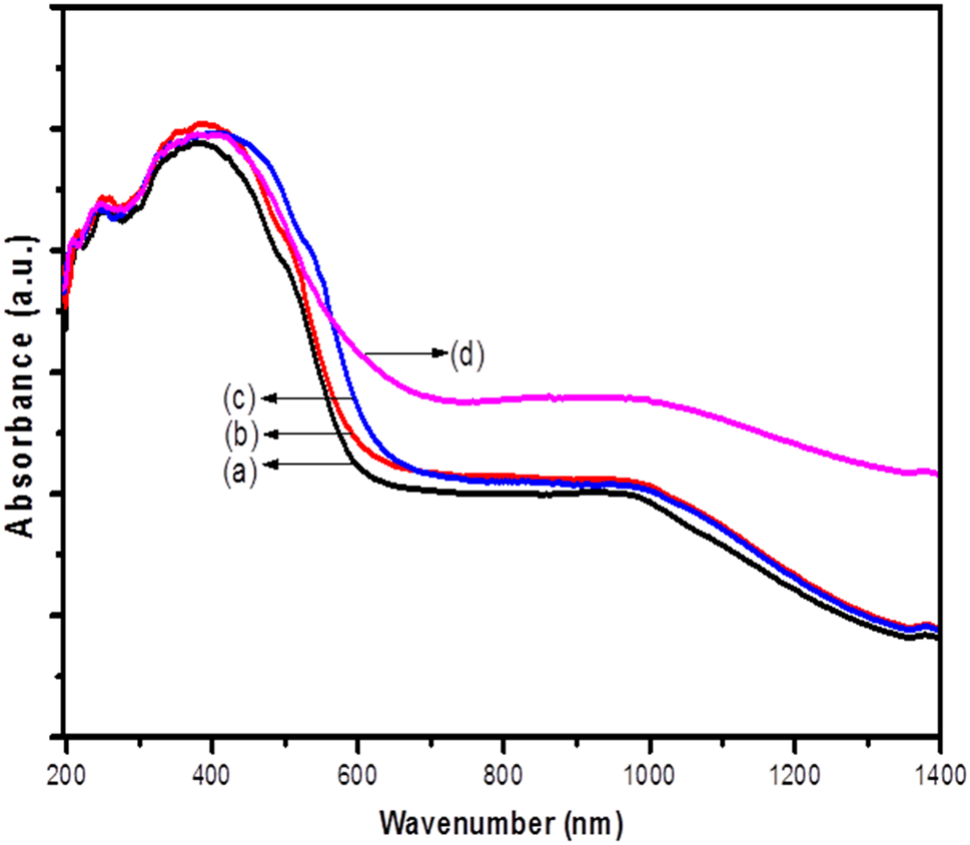
UV–Vis absorbance spectra of (**a**) Fe_3_O_4_–FeVO_4_ and xRGO/Fe_3_O_4_–FeVO_4_ at (**b**) 5%, (**c**) 10%, (**d**) 15% wt.% of RGO.

DRS spectra of Fe_3_O_4_–FeVO_4_ and xRGO/Fe_3_O_4_–FeVO_4_ with different RGO contents are depicted in Fig. 5. As depicted in Fig. 5, the samples showed strong absorption in the visible region. The absorbance threshold of Fe_3_O_4_–FeVO_4_ appeared nearly at 705 nm. However, the absorption in the visible region (strong red-shift) improved largely in the xRGO/Fe_3_O_4_–FeVO_4_ comparing with Fe_3_O_4_–FeVO_4_ indicating improving the optical properties Fe_3_O_4_–FeVO_4_ when composed with RGO^45^. The band gap energy (E_g_) was calculated using Tauc’s equation:

$$ \upalpha hv = A \left( {hv - E_{g} } \right)^{n} $$

where υ is the wavenumber, h is Planck constant, α is absorption coefficient, E_g_ is the energy band gap and A is a constant^9,29^. From the plot of (αhυ)^1/2^ versus photon energy (eV) as shown in Fig. 3S, the band gap energies (E_g_) of the prepared samples were calculated and the resulted values are listed in Table 1. As shown in Table 1, the band gap energy of Fe_3_O_4_–FeVO_4_ is 2.06 eV whereas the E_g_ of xRGO/Fe_3_O_4_–FeVO_4_ were narrowed from 2.04 of sample with 5%wt.% of RGO to 1.97 of sample with 15wt.% of RGO. The reducing in the E_g_ of xRGO/Fe_3_O_4_–FeVO_4_ can attribute to the effect and overlap of different factors. New orbitals can formed in the xRGO/Fe_3_O_4_–FeVO_4_ due to the electronic interactions between RGO and Fe_3_O_4_–FeVO_4_ leading narrowing in the E_g_ of xRGO/Fe_3_O_4_–FeVO_4_^25,46^. Another reason attributes to increase the Vo in the nanocomposite after the addition of RGO which contributes in the narrowing of E_g_ of Fe_3_O_4_–FeVO_4_ due to creation of intermediated states of energy levels in the E_g_ of xRGO/Fe_3_O_4_–FeVO_4_ causing narrowing in the E_g_^47–49^.

The creation of intermediated energy states and new molecular orbitals in the xRGO/Fe_3_O_4_–FeVO_4_ improved the absorption in the visible region and also contributed in retarding the recombination of photocarriers charges leading improving the photoactivity of xRGO/Fe_3_O_4_–FeVO_4_ under solar light^46^.

### PL spectra

**Figure 6:**
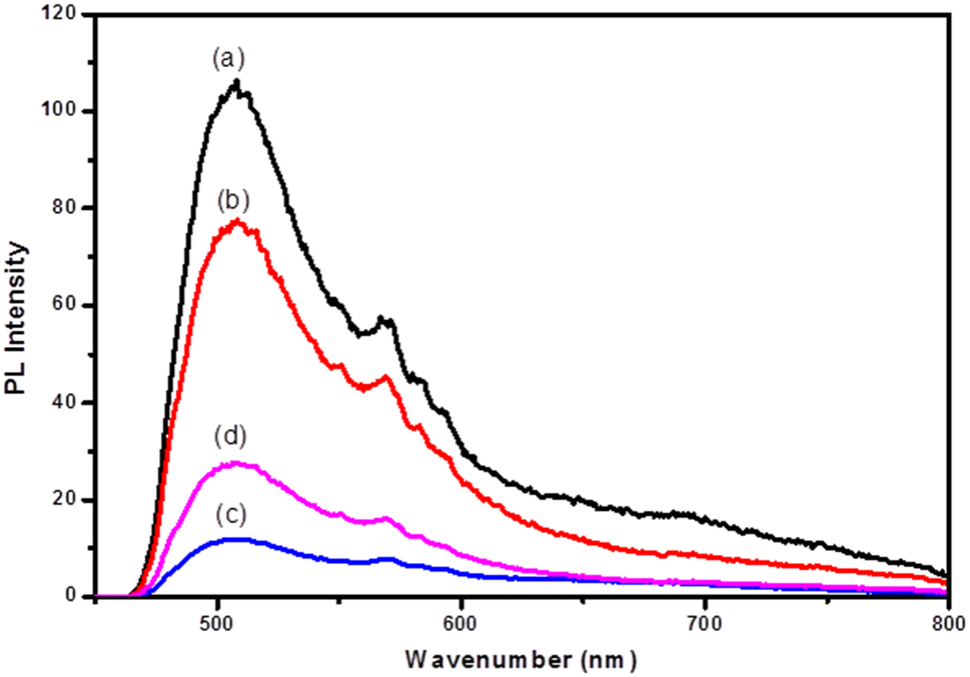
PL spectra of (**a**) Fe_3_O_4_–FeVO_4_ and xRGO/Fe_3_O_4_–FeVO_4_ at (**b**) 5%, (**c**) 10%, (**d**) 15% wt.% of RGO.

The photoluminescence spectra (PL) of Fe_3_O_4_–FeVO_4_ and xRGO/Fe_3_O_4_–FeVO_4_ are displayed in Fig. 6. All the samples displayed similar PL spectra. Figure 6 illustrates that the emission peaks intensities of xRGO/Fe_3_O_4_–FeVO_4_ decreased noticeably compared to the Fe_3_O_4_–FeVO_4_, denoting the retardation of charges carriers (e–h) recombination after the addition of RGO. On the other hand, Fig. 6 showed that the 10%RGO/Fe_3_O_4_–FeVO_4_ has the lowest intensity indicating the sample with 10 wt.% of RGO separated the photogenerated carriers effectively. The enhancing in the suppression of photocarriers of xRGO/Fe_3_O_4_–FeVO_4_ resulted from the role of RGO which acted as efficient electrons trapping by creating of new defects or vacancies within the Fe_3_O_4_–FeVO_4_ leading to enhancing the lifetime of photocarriers^50^. However, increasing the RGO amount beyond 10 wt.% accompanied by increasing the PL emission intensity (Fig. 6) indicating the opposite role of RGO where played as recombination centers and consequently accelerated the recombination rate of charges in the xRGO/Fe_3_O_4_–FeVO_4_^20,28,46^.

### Photocurrent analysis

The transient photocurrent response of the prepared samples was determined under visible light^51^. Figure 4S exhibits stable and reversible photocurrent densities of the prepared catalysts after several runs of on and off. Also, Fig. 4S displays that the 10%RGO/Fe_3_O_4_–FeVO_4_ showed the highest density comparing to other samples. Based on these results, the photocurrent density of the prepared catalysts strongly depends on the RGO content. These observations confirm the role of RGO in retardation the recombination and improving the transfer of photogenerated charges^52^. However, a reduction in the photocurrent response was observed after increasing the content of RGO to 15 wt.% indicating the opposite role of RGO as described above which in turn reduce the density of photocurrent.

### Photocatalytic performance

#### Photodegradation studies

**Figure 7:**
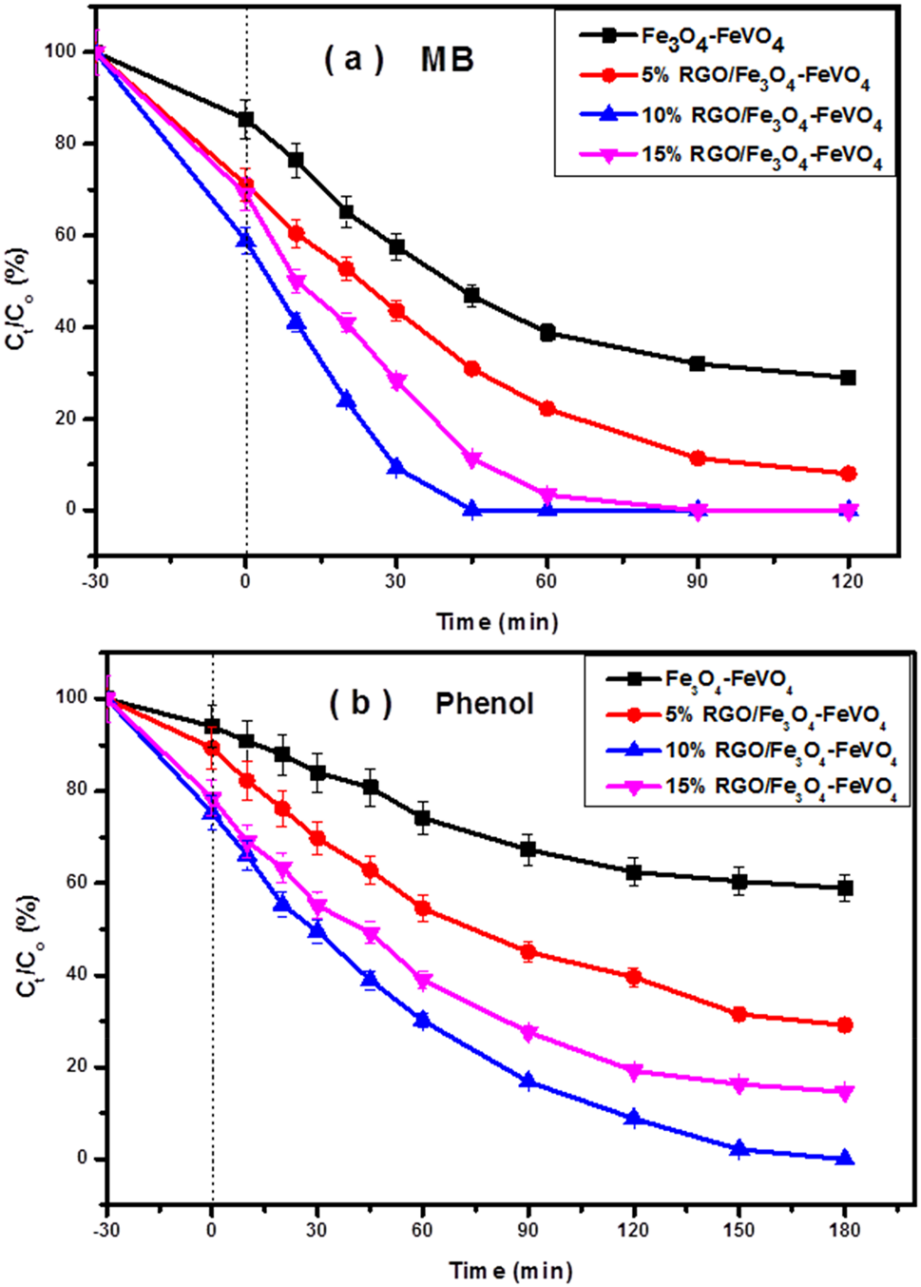
Photocatalytic degradation of (**a**) MB and (**b**) phenol over of Fe_3_O_4_–FeVO_4_ and xRGO/Fe_3_O_4_–FeVO_4_ nanocomposites versus irradiation time with error bars.

The photocatalytic performances of the prepared samples were evaluated by the photodegradation of MB, phenol and BG under sunlight irradiation as displayed in Fig. 7 and Fig. 5S, respectively. No photoreaction was observed in absence the catalyst or light source. Comparing with Fe_3_O_4_–FeVO_4_, the xRGO/Fe_3_O_4_–FeVO_4_ showed the highest photocatalytic activity against of MB, phenol and BG. Also, the sample with 10 wt.% of RGO displayed the highest photoactivity compared with other samples. These results indicate that the addition of RGO enhanced the photocatalytic activity of Fe_3_O_4_–FeVO_4_ by enhancing the suppression of the photogenerated charges recombination which in turn improved the photoactivity of Fe_3_O_4_–FeVO_4_ under sunlight irradiation^46^.

The total effect of RGO in improving and reducing the photoactivity of Fe_3_O_4_–FeVO_4_ can attributed to the following reasons: the improving resulted due to the effect of RGO in acceleration the transition of electrons to the catalyst surface leading inhibition of the photogeneration charges recombination^20^. Also, the addition of RGO facilitated the transition of electrons from VB to CB due to creation of new levels of energy through the E_g_ of Fe_3_O_4_–FeVO_4_ which in turn reduced the required energy for this transfer^53^. On the other hand, the reduction in the photoactivity of Fe_3_O_4_–FeVO_4_ in the existence of high amount of RGO resulted from the shielding effect of RGO which restrained the absorption of incident photons to arrival at the active sites on the surface of Fe_3_O_4_–FeVO_4_^20^. Moreover, increasing the amount of RGO acted as recombination centers of electrons and holes pairs^20,28,46^.

According to the Langmuir–Hinshelwood kinetics model, the photodegradation process of MB, phenol and BG over Fe_3_O_4_–FeVO_4_ and xRGO/Fe_3_O_4_–FeVO_4_ using the following formula^28^:

$$ \ln \left( {\frac{{{\text{C}}_{{\text{o}}} }}{{{\text{C}}_{{\text{t}}} }}} \right) = {\text{kt}} $$

where k is the photodegradation rate constant, C_o_ and C are the original concentration and concentration of pollutant at time t. The linear approximation of the kinetics equation is shown in Fig. 6S. The apparent values of k and correlation coefficient (R^2^) were calculated and listed in Table 2. The values of R^2^ indicated that the degradation of MB, phenol and BG follows pseudo 1st order kinetics. Also, Table 2 shows the values of k increased with increasing the RGO content and the sample with 10 wt.% of RGO displayed the highest photodegradation rate compared with other tested photocatalysts.

**Table 2:** Correlation coefficients and rate constants for MB, phenol and BG photodegradation.

Samples	MB		Phenol		BG	
	K_1_	R^2^	K_1_	R^2^	K_1_	R^2^
Fe_3_O_4_–FeVO_4_	0.01428	0.99766	0.00592	0.99318	0.01122	0.98814
5%RGO/Fe_3_O_4_–FeVO_4_	0.02304	0.99862	0.01061	0.99643	0.01749	0.99626
10%RGO/Fe_3_O_4_–FeVO_4_	0.0601	0.99959	0.02257	0.02257	0.04989	0.99846
15%RGO/Fe_3_O_4_–FeVO_4_	0.04161	0.9986	0.01599	0.01599	0.03295	0.99875

Figure 7S illustrates the values of %TOC of MB, phenol and BG degradation over 10%RGO/Fe_3_O_4_–FeVO_4_. The results illustrated that the mineralization of MB, phenol and BG after 180 min was 92.8%, 85.3% and 99.9%, respectively. Comparing these results with the photodegradation results we found the values of %TOC were lower than the photodegradation values. This indicates to existence of some un-degraded intermediates (colorless). However, with increasing the irradiation time to 360 min, the %TOC values increased sharply to achieve 100% of mineralization of both MB, phenol and BG.

Table 1S displays comparison between the photocatalytic activity of our samples that obtained in this work and that found in other literatures^17,19,20,22,23,25,27,54–58^. As shown in Table 1S the 10%RGO/Fe_3_O_4_–FeVO_4_ showed the highest photoactivity for photodegradation of organic pollutants under solar light. Also, our samples showed high visible light absorption, photogenerated charges separation and reusability. Moreover, in this work, the reduction of graphene oxide was performed effectively without using chemical reduction agents by green reduction method and during short time (2 h) comparing to other literatures.

#### Photocatalytic mechanism

**Figure 8:**
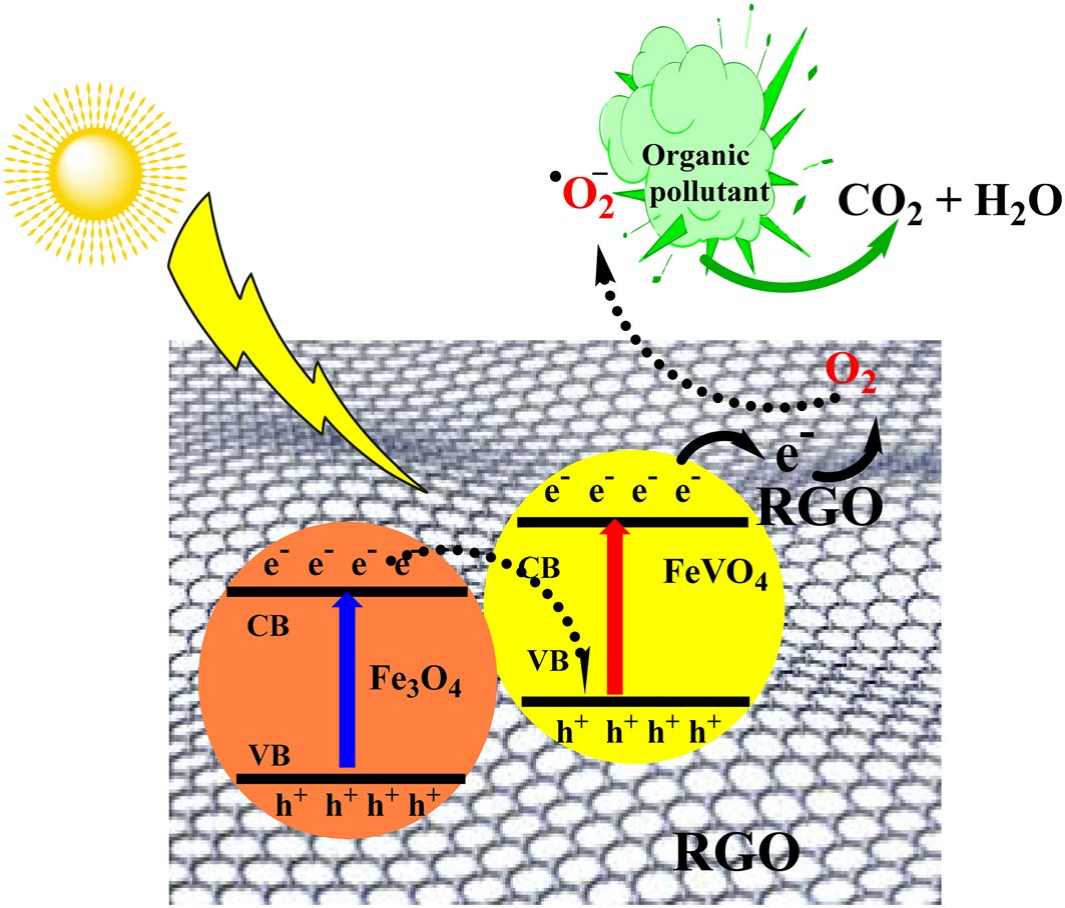
Postulated mechanism of electron transfer in xRGO/Fe_3_O_4_–FeVO_4_ nanocomposites.

To understand the photodegradation mechanism of MB, the radical scavengers were added during the photodegradation of MB. Figure 8S shows effect the addition of scavengers on the degradation of MB over 10%RGO/Fe_3_O_4_–FeVO_4_ sample. As shown in Fig. 8S, the addition of BQ (^·^O_2_^**−**^ radical) was accompanied with sharply reduction in the degradation of MB while the addition of Na_2_EDTA (h^+^ radical) and IPA (^·^OH radical) accompanied with high suppression for Na_2_EDTA and venial reduction for IPA. These results imply that the ^·^O_2_^**−**^ played the main role in degradation of MB while the ^·^OH played the minor role. This indicates that the ^·^O_2_^**−**^ is the more active radical contributed in the degradation of MB.

From the radical scavengers results, the possible mechanism of the degradation of MB, phenol and BG over xRGO/Fe_3_O_4_–FeVO_4_ was suggested as a direct Z-scheme as proposed in Fig. 8. In this mechanism, when the xRGO/Fe_3_O_4_–FeVO_4_ absorbed the sunlight illumination, the incident photons promoted transfer of electrons (e^**–**^) to the CB of FeVO_4_ and the holes (h^**+**^) accumulated in the VB of Fe_3_O_4_ [Eqs. (3), (4)]. The photoinduced e^**–**^ on the CB of Fe_3_O_4_ can transfer to the VB of FeVO_4_, and then quickly recombine with the h^**+**^ of FeVO_4_ were achieved, which is favorable for higher separation rate of e^**–**^-h^**+**^ pairs of the single photocatalyst and higher redox potential^59,60^. In this case, the e^**–**^ accumulated in CB of FeVO_4_ are taken by the RGO and then reduce O_2_ into ^·^O^2–^ [Eqs. (5), (6)], while the h^**+**^ in VB of Fe_3_O_4_ VB are more positive potential and has sufficient oxidation capacity to oxidize OH^**–**^ into ^·^OH or react with pollutants molecules directly [Eqs. (7–9)]^59–63^. These results imply the contribution of generated radicals in the degradation of MB and phenol to gives CO_2_ and H_2_O.

3 $$ {\text{Fe}}_{3} {\text{O}}_{4} + {\text{h}}\upnu \left( {{\text{photon}}} \right) \to {\text{e}}^{ - } + {\text{Fe}}_{3} {\text{O}}_{4}^{*} \left( {{\text{h}}^{ + } } \right) $$

4 $$ {\text{FeVO}}_{4} + {\text{e}}^{ - } \to {\text{FeVO}}_{4} \left( {{\text{e}}^{ - } } \right) $$

5 $$ {\text{FeVO}}_{4} \left( {{\text{e}}^{ - } } \right) + {\text{RGO}} \to {\text{RGO}}\left( {{\text{e}}^{ - } } \right) $$

6 $$ {\text{RGO}}\left( {{\text{e}}^{ - } } \right) + {\text{O}}_{2} \to {\text{RGO}} + ^{ \cdot } {\text{O}}_{2}^{ - } $$

7 $$ {\text{Fe}}_{3} {\text{O}}_{4}^{*} \left( {{\text{h}}^{ + } } \right) + {\text{H}}_{2} {\text{O}} \to {\text{H}}^{ + } + {\text{OH}}^{ - } $$

8 $$ {\text{Fe}}_{3} {\text{O}}_{4}^{*} \left( {{\text{h}}^{ + } } \right) + {\text{OH}}^{ - } \to^{ \cdot } {\text{OH}} $$

9 $$^{ \cdot } {\text{O}}_{2}^{ - } + {\text{h}}_{{{\text{VB}}}}^{ + } +^{ \cdot } {\text{OH}} + {\text{Organic}}\;{\text{pollutants}} \to {\text{CO}}_{2} + {\text{H}}_{2} {\text{O}} $$

#### Reusability study

**Figure 9:**
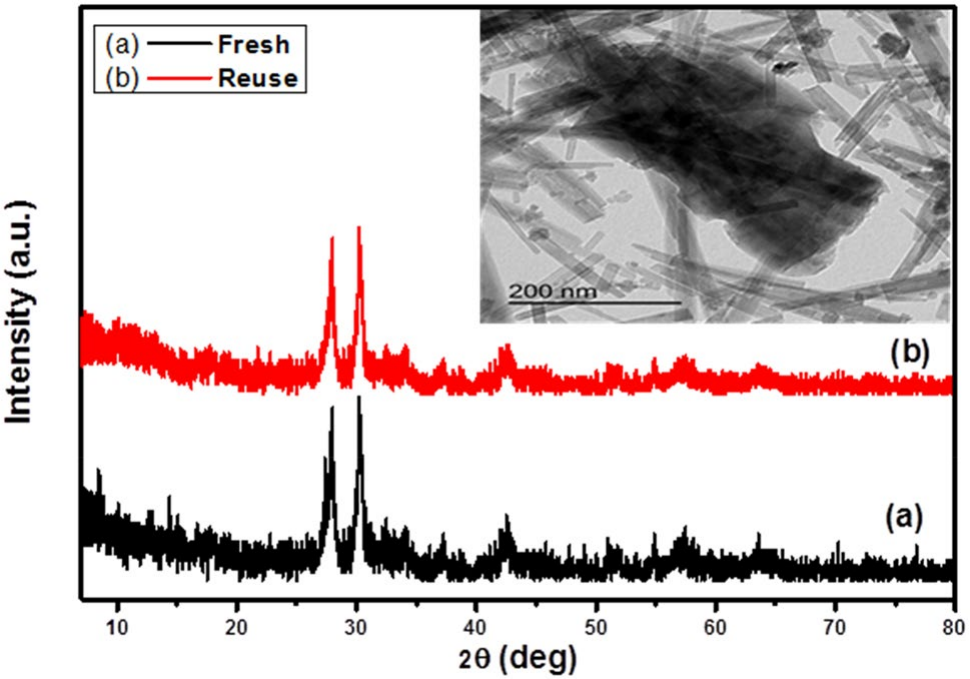
Effect of reuse on the structural properties of 10% rGO/FeVO_4_ nanocomposites.

The reusability and stability of 10%RGO/Fe_3_O_4_–FeVO_4_ were studied. Figure 9S displays the degradation of MB, phenol and BG after four cycles (runs) under the same conditions. The catalyst powder was separated from the reaction mixture after each run and soaked in ethanol for 1 h. Then, the powder was washed with water and finally dried at 100 °C for 8 h^30^. The obtain results illustrated no significant decline in photodegradation activity of 10%RGO/Fe_3_O_4_–FeVO_4_ was observed after five runs. To investigate the effect of reused times on the structural properties of catalyst, the 10%RGO/Fe_3_O_4_–FeVO_4_ was investigated by the XRD and TEM techniques before and after reuse as shown in Fig. 9 and the results showed no changes were observed in the structural properties of 10%RGO/Fe_3_O_4_–FeVO_4_ indicating the excellent reusability and sustainability of the prepared photocatalysts.

## Conclusion

Fe_3_O_4_–FeVO_4_ and xRGO/Fe_3_O_4_–FeVO_4_ were successfully prepared by the hydrothermal method. The results confirmed successfully reduction GO to RGO by green method. The UV–Vis results showed improving of the absorption in the visible region and enhancing of the photogenerated charges separation of after the addition of RGO. The samples showed excellent degradation of MB, phenol, BG after the addition of RGO under sunlight illumination and the sample with 10 wt.% of RGO exhibited the highest photodegradation efficiency. The TOC analysis illustrated that the MB and phenol completely mineralized after 360 min. The results of scavenger tests showed that the ^·^O_2_^**−**^ played the main role in oxidant of MB. The kinetic studies results illustrated that the degradation of MB and phenol follows the pseudo 1st order kinetics. The prepared samples showed excellent reusability after five runs without significant reduction in the photoaactivity. Based on these results, we can be concluded that the xRGO/Fe_3_O_4_–FeVO_4_ are suitable and convenient material for treatment of organic pollutants and industrial effluent.

## Supplementary Information

Supplementary Information.
