# Supplementary material for: One-step preparation of RGO/Fe3O4–FeVO4 nanocomposites as highly effective photocatalysts under natural sunlight illumination
Source: Sci Rep. 2022 Apr 21;12:6565. doi: 10.1038/s41598-022-10542-z (PMC9023601; doi:10.1038/s41598-022-10542-z)
Supplement: Supplementary file 1 — Supplementary Information. [file 41598_2022_10542_MOESM1_ESM.docx]

**One-step preparation of RGO/Fe_3_O_4_-FeVO_4_ nanocomposites as highly effective photocatalysts under natural sunlight illumination**

**Qana A. Alsulami ^a^,*, A. Rajeh ^b^, Mohammed A. Mannaa ^c^, Soha M. Albukhari ^a^, Doaa F. Baamer ^a^**

^a^ Chemistry Department, Faculty of Science, King Abdulaziz University, Jeddah, Saudi Arabia.

^b^ Physics Department, 1q2Faculty of Science, Amran University, Yemen.

^c^ Chemistry Department, Faculty of Science, Amran University, Yemen.


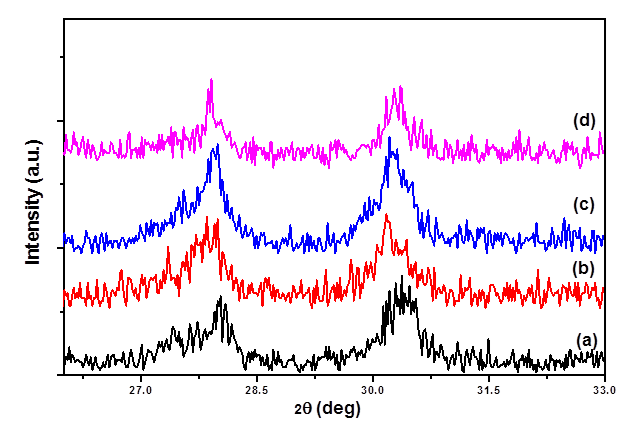
Fig.1S. XRD patterns of (a) Fe_3_O_4_-FeVO_4_ and (b) 5%, (c) 10%, (d) 15%xRGO/Fe_3_O_4_-FeVO_4_ nanocomposites


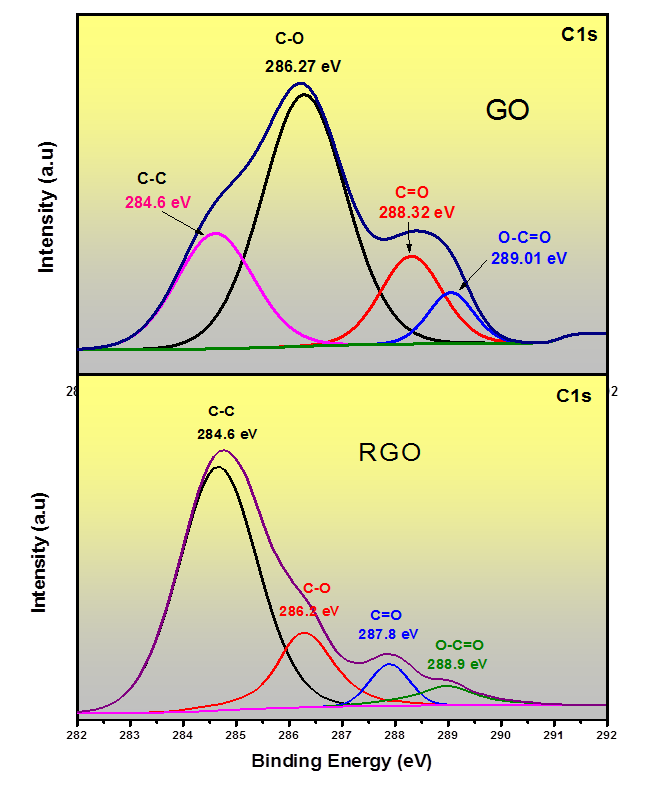


Fig.2S: XPS spectra of GO and RGO.

**
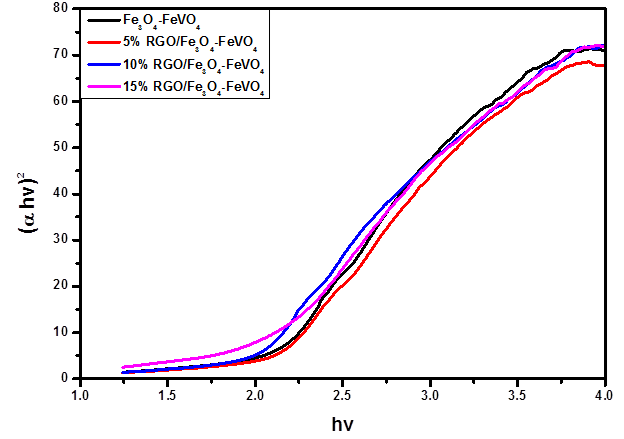
**

Fig.3S. Tauc’s plot for optical band gap calculation of Fe_3_O_4_-FeVO_4_ and xRGO/Fe_3_O_4_-FeVO_4_

**
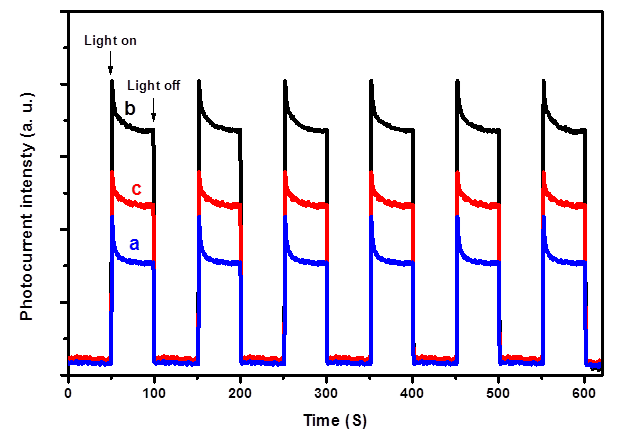
**

Fig.4S. Photocurrent response of (a) Fe_3_O_4_-FeVO_4_ (b) 10%RGO/Fe_3_O_4_-FeVO_4_ and (c) 15%RGO/Fe_3_O_4_-FeVO_4_

**
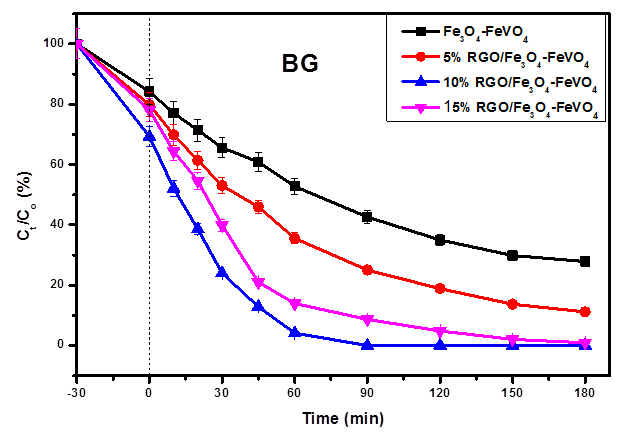
**

Fig.5S. Photocatalytic degradation of BG over of Fe_3_O_4_-FeVO_4_ and xRGO/Fe_3_O_4_-FeVO_4_nanocomposites vs. irradiation time with error bars.


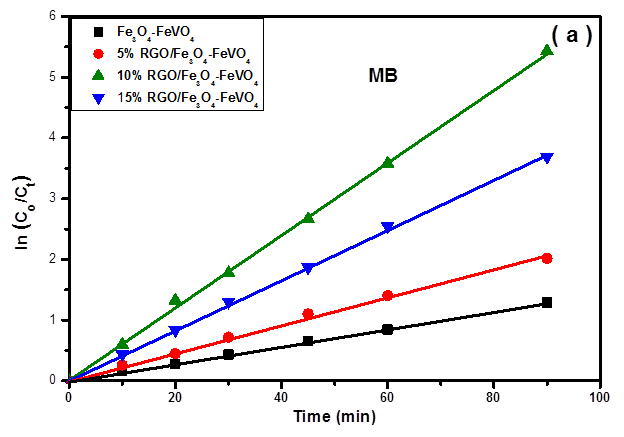

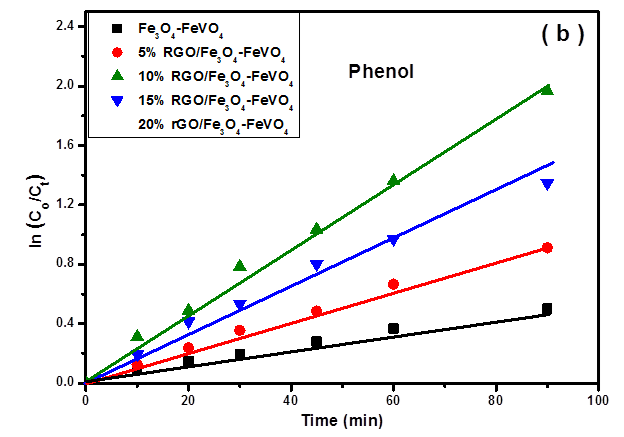


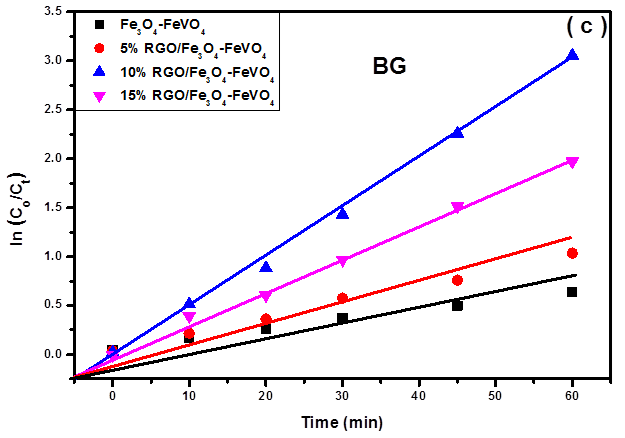


Fig. 6S. The pseudo first-order kinetic of photodegradation of (a) MB and (b) phenol (c) BG over of Fe_3_O_4_-FeVO_4_ and xRGO/Fe_3_O_4_-FeVO_4_nanocomposites

**
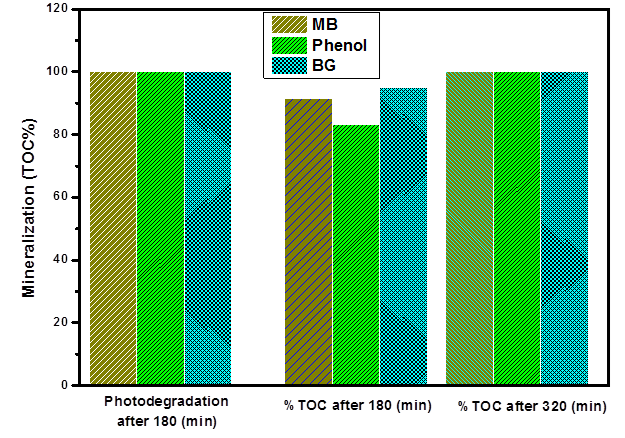
**Fig.7S: TOC% removal and photodegradation of MB, phenol and BG vs. time


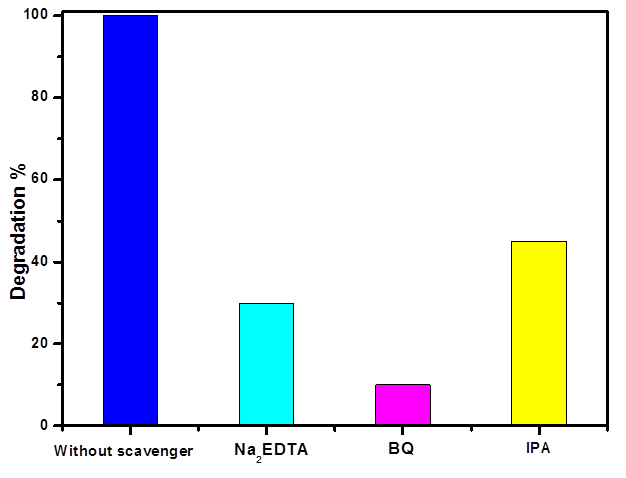
Fig.8S: Photodegradation of MB over 10%RGO/Fe_3_O_4_-FeVO_4_ in the absence and presence of different scavengers under similar reaction conditions.


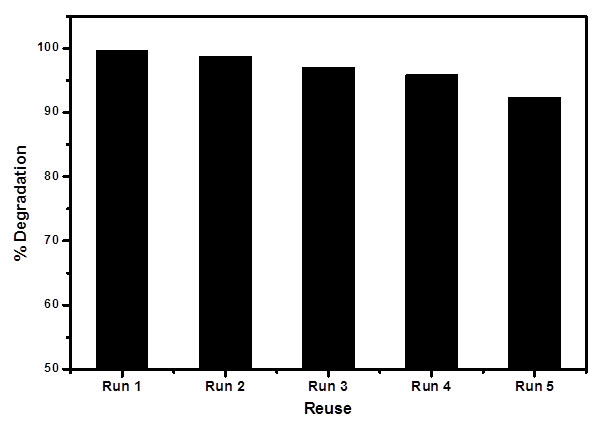


a


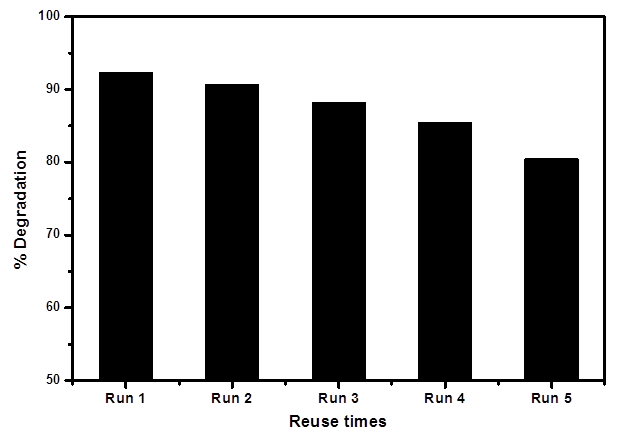


b

.

c


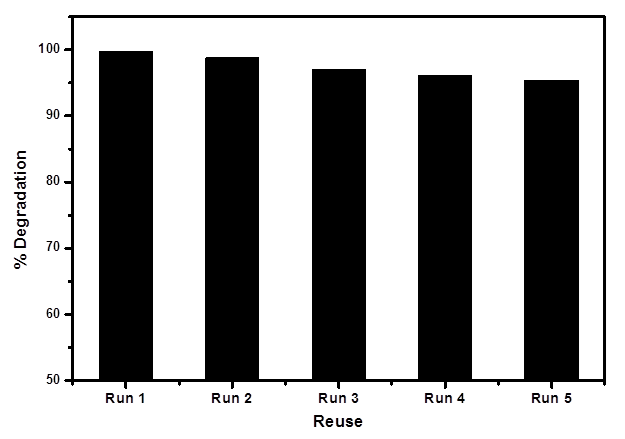


Fig. 9S. Effect of reuse times of 10%RGO/Fe_3_O_4_-FeVO_4_photocatalyst on the photocatalytic degradation of (a) MB and (b) Phenol (c) BG.

Table 1S: Element analyses of 10%RGO/Fe_3_O_4_-FeVO_4_ nanocomposites.

| Elements | Weight% |
| --- | --- |
| C | 50.71 |
| O | 23.65 |
| V | 16.63 |
| Fe | 9.01 |

Table 2S: Comparison of the photodegradation performances of different organic pollutants by different photocatalysts.

| **Catalyst** | **Type of pollutant** | **Light source** | **Time, (min.)** | **Efficiency (%)** | **Ref.** |
| --- | --- | --- | --- | --- | --- |
| **RGO/Fe_3_O_4_-FeVO_4_** | MB | Sunlight | 60 | 100% | This  work |
|  | Phenol |  | 150 | 100% |  |
|  | BG |  | 90 | 100% |  |
| **rGO-FeVO_4_,**  **(0.05 gm)** | MG | Sunlight | 120 | 100% | [27] |
|  | Phenol |  | 180 | 92.3% |  |
|  | MB |  | 60 | 100% |  |
|  | RhB |  | 90 | 100% |  |
| **rGO-FeVO_4_,**  **(0.12 gm)** | MB | Visible | 180 | 80.5 | [54] |
| **FeVO_4_/Bi_7_O_9_I_3_**  **(0.1 gm)** | MB | visible | 360 | 81.3 | [23] |
|  | RhB |  | 360 | 98.9% |  |
|  | MO |  | 360 | 94.9% |  |
| **BiVO_4_/FeVO_4_@rGO (0.3 gm)** | tetracycline | visible | 90 | 93 | [20] |
| **ZnO/FeVO_4_ /H_2_O_2_ /pH5.5** | Sodium dodecyl sulfate | UV | 45 | 99% | [22] |
| **BiVO_4_/FeVO_4_ (0.2gm)** | Metronidazole | visible | 90 | 90% | [17] |
| **RGO-FeWO_4_/Fe_3_O_4_ (0.2 gm)** | RhB | visible | 240 | 35% | [25] |
| **NRGO-CoWO_4_-Fe_2_O_3_ (0.2 gm)** | MB | visible | 120 | 98.23 | [55] |
| **FeVO_4_:Zn^2+^,**  **(0.03 gm)** | MB | Visible | 180 | 99 | [19] |
| **FeVO_4_:Mn^2+^,**  **(0.03 gm)** | MG | Visible | 180 | 98 | [19] |
| **FeVO_4_-IL/H_2_O_2_,**  **(0.1 gm)** | RhB | Visible | 120 | 95 | [57] |
| **Bi_2_O_3_/FeVO_4_**  **(0.05 gm)** | MG | Visible | 240 | 88.7 | [58] |
